# Development and initial validation of an instrument to measure novice nurses’ perceived ability to provide care in acute situations – PCAS

**DOI:** 10.1186/s12912-020-0406-3

**Published:** 2020-02-17

**Authors:** Anders Sterner, Emma Säfström, Lina Palmér, Nerrolyn Ramstrand, Magnus Andersson Hagiwara

**Affiliations:** 1 Faculty of Caring Sciences, Work Life and Social Welfare, University of Borås, SE-501 90 Borås, Sweden; 2 Department of Medical and Health Sciences, Linköping University, 581 83 Linköping, Sweden; 3 CHILD research group, Department of Rehabilitation, School of Health Sciences, Jönköping University, 551 11 Jönköping, Sweden; 4 Centre for Prehospital Research, Faculty of Caring Science, Work Life and Social Welfare, University of Borås, 501 90 Borås, Sweden

**Keywords:** Nursing, Education, Educational measurement, Psychometrics, Self report, Acute

## Abstract

**Background:**

Novice nurses need to be better prepared to provide care in acute situations. There is currently no validated scale specifically measuring nurses’ perception of their ability to provide care in acute situations. The aim of this study was to develop and examine the psychometric properties of a scale that measures novice nurses self-reported perception of ability to provide care in acute situations.

**Method:**

Development and test of the psychometric properties of the Perception to Care in Acute Situations (PCAS) scale. Items were generated from interviews with novice nurses (*n* = 17) and validated using opinions of an expert panel and cognitive interviews with the target group.

Two hundred nine novice nurses tested the final scale. Exploratory factor analysis (EFA) was used to test construct validity, item reduction and underlying dimensions between the measured variables and the latent construct.

**Result:**

The PCAS scale contains 17 items grouped into three factors. EFA demonstrated a clean three factor logic construct solution with no cross-loadings, high correlation for the total scale in both Cronbach’s alfa 0.90 and ordinal alpha 0.92.

**Conclusions:**

The PCAS scale has proven to have acceptable validity. The factors,” confidence in provision of care”, “communication” and “patient perspective” are likely to be important aspects of providing care in acute situations. Additional testing of the PCAS is needed to conclude if it is sensitive enough to evaluate interventions aimed at improving novice nurses competence and suitable as a guide for reflection for novice nurses.

## Background

Novice nurses typically receive a comprehensive theoretical education but there are indications in Western culture that they are poorly prepared for practical clinical work [1]. A growing number of interventions have been suggested and implemented to address this alleged lack of work preparedness. These include simulation training in nursing education [2, 3], internship/residency programs, standardized nurse orientation, simulation-based graduate programs and mentorship/preceptorship for new nurses [4]. Gradate nurses entering practice today encounter a health care system characterized by workforce shortages, economic restrictions, complex organizations and increasing patient acuity. In addition, the body of knowledge is increasing exponentially along with development of new technologies that can influence practice [5]. This raises debate as to whether it is realistic to assume that novice nurses have the ability to work independent when entering the profession [6].

Benner [7] has described the difference between experienced nurses and novice nurses in complex nursing practice. She suggests that in the acquisition and development of skills nurses pass through five levels of proficiency which reflect changes in two general aspects of skill performance; the use of concrete experience (not mere passage of time) rather than abstract principles and a change in the perception and understanding of demanding situations. These changes mean that nurses see situations as less of a compilation of equally relevant parts and more as a complete whole in which only certain parts are relevant.

Acute care situations represent a particular situation in which novice nurses feel unprepared and experience a sense of overwhelming responsibility [8]. Despite being novice or experienced, the nurse is expected to respond as frequently and competent in acute situations so as to prevent adverse events and improve patient outcome [9] . The concept ‘acute’ is somewhat unclear even if it is commonly used in both clinical and theoretical contexts. The semantic definition of ‘acute’ is sharp and intense. Synonyms also suggest something that is temporal, rapid or swift [10]. In a study describing the acute situation as a phenomenon from the perspective of novice nurses, acute situations have been considered to include significantly more than just the state of a clinically deteriorating patient. Acute situations involve situations where something happens suddenly, time is insufficient, one’s own competence is perceived as inadequate, responsibility is overwhelming, organizational deficiencies become apparent and when there are challenges in interpersonal relations [11]. A number of issues have been identified which influence novice nurses’ ability to provide care in acute situations. Studies have indicated that personal experience of managing acute situations [9, 12, 13] and educational programs that integrate theory and practice [13, 14] are considered by nurses themselves to be key facilitators. Studies also suggests that simulation can promote the ability to care in acute situations [13, 15, 16].

Outcomes of interventions aimed at improving nurses’ ability to provide care are typically evaluated using self-report measures [2–4]. There are a variety of self-report measurement scales that have been developed to evaluate nurses’ competence and ability in different areas of nursing. Measures of competence are often developed from national goals or guidelines [17, 18] or on the basis of theoretical framework [19]. Other measures focus on specific skills considered essential in nursing [20, 21].

As described earlier, initiatives have been undertaken to better prepare novice nurses for practice and specifically for managing acute situations. To the authors’ knowledge however, there is currently no measurement scale constructed with the goal of evaluating novice nurses’ perceptions of their ability to care in situations which they themselves would describe as acute. Such a scale would facilitate evaluation of educational interventions and serve as a guide for novice nurses to reflect on areas they feel are problematic. The aim of this study was to develop and examine the psychometric properties off a scale that measures novice nurses self-reported perception of their ability to care in acute situations.

## Methods

### Scale development

The construct to be measured by the Perception to Care in Acute Situations (PCAS) scale is, novice nurses’ perceived ability to care in acute situations. For the purpose of development, a novice nurse was defined as an individual who has worked in the profession for less than 1 year. The demarcation of less than 1 year of working experience was based upon Benner’s novice to expert theory [7].

The term ‘ability’ was considered to reflect perceived performance on a variety of tasks, described in the specific items within the scale and considered as a requirement of nurses who provide care in acute situations. Care was defined as actions taken by the nurse with the aim of improving health and reducing suffering [22]. Acute situations were considered as those perceived by novice nurses themselves as being acute. This could include situations in which something happens suddenly and the care situation changes or the perception that there is insufficient time in relation to the tasks that must be performed. A common example of an acute situation would be when the patient suffers from a sudden illness [11].

#### Item generation

An inductive approach was used to generate potential items for the PCAS scale [23, 24]. Items were based upon interviews (*n* = 17) conducted in two previously published studies with novice nurses. These studies investigated participants’ perception of acute situations and identified factors which are considered necessary in developing the ability to care in acute situations [11, 13].

The initial pool of items for the PCAS scale (*n* = 40) was generated by the first author and later discussed within the research team. Use of a team to develop items took advantage of that people articulate similar ideas in diverse ways [25]. During discussions with the research team, several items were deleted, rewritten and added. When consensus was reached, the total number of items was 45. Eleven of these were background questions while the remaining 34 items addressed ability to care in acute situations. These 34 items were subsequently assessed for content validity.

#### Content validity

Content validity of items was assessed by a group of experts and by a group representing the target population (novice nurses). The expert panel was contacted by the first author via e-mail and comprised of four individuals who were recruited on the basis that they had well documented clinical experience and/or research experience on the topic [26]. Experts were all registered nurses and had knowledge of acute care and nursing education. They varied in sex, age, type of clinical experience and clinical specialization. The panel consisted of a nursing program director with a doctoral degree from a university in Sweden, a lecturer in nursing with responsibility for a course in acute care assessment, a senior lecturer with doctoral degree specializing in emergency care and an experienced registered nurse employed within a medical ward and responsible for introducing novice nurses to the ward. The panel of experts were requested to independently comment on the relevance of specific items and the structure of the scale. They were asked to e-mail their responses to the first author.

The group of novice nurses representing the target population were recruited by the first and second authors on the basis that they worked in somatic care in different regions of Sweden. They comprised of one man working in an accident and emergency ward and one woman working on a medical acute care ward. For these individuals cognitive interviewing was used as a measure of content validity [23]. We used a combination of thinking-aloud (were subjects are instructed to “think aloud” when answering the questions) and probing (were the interviewer asks for information relevant to the question or answer given) [27]. The first interview was based upon the first draft of the scale while the second was based upon the revised version.

After receiving feedback from the expert group and following the cognitive interviews, items were revised for increased clarity and face validity. When modifications had been made, a second cognitive interview was performed with one novice nurse. The research team then met to discuss each item and reach consensus about the final items to include in the scale. Consensus was reached on 33 items. All 33 required a response on a four-point Likert scale. An even 4-point scale was chosen so as to force respondents to clearly express their perceived ability to care in acute situations [24]. The response categories ranged from strongly disagree to strongly agree and very poor to very good.

All items were entered into a web-based survey program (Sunet Survey, version 4.3.9.5, Artisan Global Media, Sweden) and a link to the questionnaire was generated to allow for anonymous login.

### Psychometric testing

#### Sample selection and recruitment

A sample of novice nurses were recruited to participate in psychometric testing of the scale. The sample included novice registered nurses who had been working for less than a year in a somatic ward at a hospital in Sweden. Nurses were recruited by contacting representatives from county and university hospitals within each of the 21 healthcare regions of Sweden. Representatives were requested to distribute an e-mail invitation to staff who met the inclusion criteria. The invitation included details of the study, information that participation was anonymous and voluntary, contact information for the researchers and a link to the web-based questionnaire. Participants were encouraged to share the link with other novice nurses who met the inclusion criteria for this study. There is no consensus among experts on how large of a sample is needed to test scales. Recommendations are however available regarding subject (respondent) to item ratios and total sample size [25]. The sample size was subsequently determined using the recommended minimum subject/item ratio of > 5:1 [28] or at least 200 respondents [23].

#### Data analysis

Descriptive and frequency statistics were used to analyze missing data, errors, demographics, frequency of endorsements and discrimination within items.

Exploratory factor analysis (EFA) was used to test the construct validity of the scale [29]. Given the ordinal nature of the data, factorability was investigated by assessing polychoric correlation [30]. Factorability was assumed if item total correlations >.30 [24], Kaiser-Meyer-Olkin (KMO) > 0.6 and Bartlett’s test of sphericity [28].

Construct validity, item reduction and underlying dimensions between the measured variables and the latent construct were assessed using EFA [29] with ordinary least squares [31]. As we expected some correlation between factors, oblique rotation was used [32].

The number of factors to retain in the model was determined using a scree plot, parallel analysis, Eigenvalue > 1 and Velicer MAP. At least three items were required within each factor [32].

Items were deleted if there were factor loadings <.32, [29, 32] or cross-loading (i.e. discrimination between factors) < .20.

Internal consistency was evaluated using polychoric corrected item - total correlations and ordinal alpha [23, 33]. For comparative purposes we also present Cronbach’s alpha ≥.70 [23]. All statistical analysis was performed in R statistical software 3.5.3 [34] with Psych package 1.8.12 [35]. and the R-studio interface version 1.2.1335.

#### Ethical considerations

This study did not involve patients, relatives or sensitive personal information. As such the study did not fall within the boundaries of the Ethical Review Act 2003:460 [36], which regulates all types of research involving humans in Sweden and was not required to be submitted for ethical approval, locally or nationally. The research was however conducted in accordance with the requirements of the Declaration of Helsinki [37]. This has been done by ensuring that participation in the study was anonymous and ensuring that it was not possible for anyone in the research team to identify any of the nurses responding to the survey. Consent to participate was assumed if nurses chose to complete the web-based questionnaire.

## Results

**Fig. 1:**
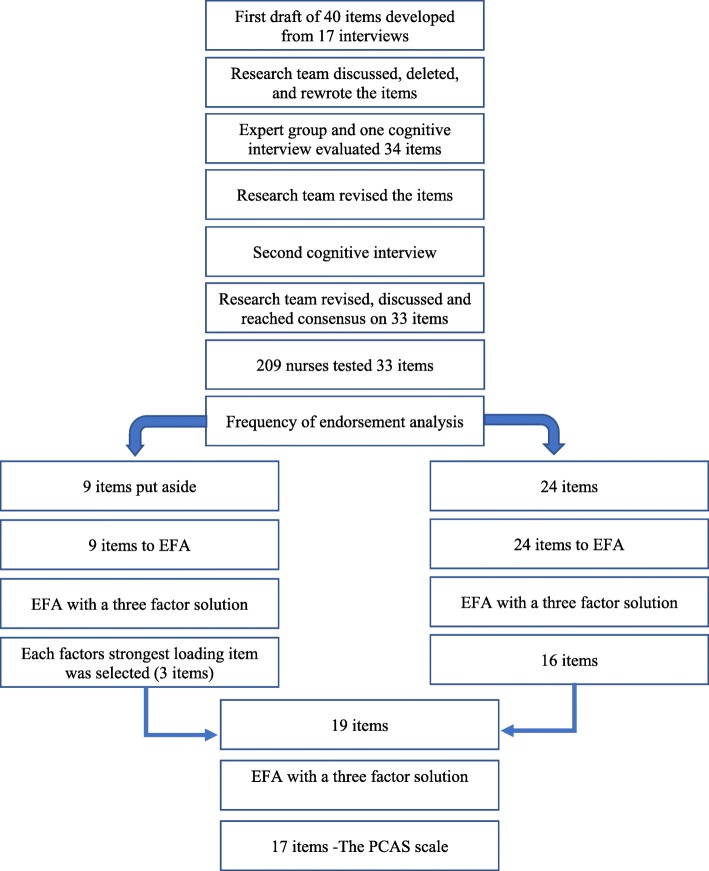
Flow chart of the PCAS development process

Representatives from 14 of the 21 healthcare regions in Sweden agreed to contact staff meeting the inclusion criteria. The sample represented 33 hospitals (both county and university hospitals) and a total of 227 participants completed the survey. No missing data was recorded as the electronic questionnaire could not be submitted if there was any missing data. Seventeen participants were excluded as they did not fulfil the inclusion criteria of having worked less than 1 year within somatic care. One additional participant was excluded as they did not indicate how many months they had been working after graduation. Demographic descriptions of the remaining 209 respondents are presented in Table 1, (Fig. 1)

**Table 1:** Respondents demographics

Variable	Value
Participants	209
Gender, n (%)	
Female	175 (83,7)
Male	33 (15,8)
Other/Don’t want to say	1 (0,5)
Median age (range)	26 (22–54)
Median months working experience (range)	6 (1–12)
Universities^a^, n	27
Experience of acute situations since graduation, n (%)	
Little	161 (77)
Much	48 (23)
Hospitals, n	33
Workplace	
Accident and emergency dep, n (%)	25 (12)
Medical, n (%)	75 (36)
Surgical, Orthopaedic, n (%)	63 (30)
Other/combination, n (%)	46 (22)

^a^Universities nurses graduated from

Frequency of endorsement of respondent categories as a measure of item discrimination (i.e. variation in respondents) showed 9 problematic items with zero endorsement in the first response category. These types of items do not improve psychometric properties [24] and were subsequently put aside but not discarded due to their theoretical importance [38]. To see the all 33 items, please refer to the supplementary table with item data description.

Correlations for the remaining set of 24 items was ≥0.40–0.73. Kaiser-Meyer-Olkin (KMO) test for sampling adequacy was >.91 and Bartlett’s test of sphericity was significant, K-squared = 129.06, df = 23, *p* = < 0.001. The scree plot for the 24 items indicated a four-factor solution while parallel analysis of polychoric correlations suggested a three-factor solution. Eigenvalue >1 indicated a four-factor solution and the Velicer MAP achieved a minimum of 0.02 with 3 factors.

We subsequently tested a four-factor solution. For every item deleted, in accordance to the pre-established criteria; factor loadings <.32, or cross-loading (i.e. discrimination between factors) < .20. a new EFA was run and the factor loading matrix was analyzed. This was done to ensure that item deletion did not change the factor structure, cross-loadings or factor loadings [28].

After deletion of numerous items, it was apparent that the four-factor solution did not fulfil the pre-established requirement of three items in each factor solution.

A three-factor model was then attempted based on the parallel analysis and Velicer MAP which had indicated three factors. The same factor model approach and predetermined rules as the four-factor model were used. This model resulted in a 16-item solution that was judged as clear and comprehensible and fulfilled all criteria.

Given that the 9 items removed during the initial analysis of response frequencies were considered to be theoretically important, an attempt was made to further analyze them in the same way as the other items. Results from the scree plot and a parallel analysis on these items indicated that a three-factor solution would be most appropriate. Therefore, we conducted an EFA with ordinary least square and oblique rotation, direct oblimin displaying the three-factor solution on the 9 items. The items with the strongest loading for each of the three factors were then added to the 16-item scale resulting in a total of 19 items.

An EFA on the 19-items resulted in a cross-loading and deletion of two items, in accordance to the pre-established criteria. Additional re-run of the EFA displayed a good three factor fit that was judged as theoretically sound, clear, comprehensible and fulfilling all criteria. The pattern matrix is presented in Table 2. Factors were named, 1/” confidence in provision of care” [10 items], 2/ “communication” [4 items] and 3/ “patient perspective” [3 items] including a total of 17 items.

**Table 2:** Pattern matrix of loadings score with oblique (oblimin) rotation (*N* = 209)

Item	Factor 1	Factor 2	Factor 3
1. I worry about providing care in acute situations	**0.75**	0.02	−0.11
2. I trust my ability to provide care in acute situations	**0.74**	0.01	−0.07
3. I have sufficient knowledge to provide care in acute situations	**0.62**	0.05	0.03
4. I estimate my general ability to provide care in acute situations	**0.73**	0.00	0.02
5. I estimate my ability to manage the demands that I place upon myself in acute situations	**0.80**	0.09	0.07
6. I estimate my ability to manage demands from my colleagues in acute situations	**0.68**	−0.04	−0.04
7. I estimate my ability to independently determine necessary actions in acute situations	**0.62**	0.12	0.07
8. I estimate my ability to independently prioritise between actions in acute situations	**0.57**	0.14	0.02
12. I estimate my ability to independently lead bedside care in acute situations	**0.55**	0.15	0.09
16. I estimate my ability to understand the individual patient’s medical needs in acute situations	**0.46**	0.09	0.18
9. I estimate my ability to take instructions over the telephone in acute situations	−0.06	**0.94**	0.01
10. I estimate my ability to carry out instructions that I have received over the phone in acute situations	0.04	**0.83**	−0.04
11. I estimate my ability to receive instructions from an attending doctor in acute situations	0.17	**0.65**	0.02
13. I estimate my ability to report a patient’s condition to a nurse in an acute situation	0.23	**0.49**	0.18
14. I estimate my ability to make patients participate in acute situations	−0.10	0.04	**0.84**
15. I estimate my ability to understand the individual patient’s care needs in acute situations	0.13	−0.08	**0.83**
17. I estimate my ability to provide information adapted to the needs of the individual in acute situations	0.02	0.23	**0.55**
Ordinal alpha: Total scale 0.92	0.90	0.88	0.81
Cronbach alpha: Total scale 0.90	0.89	0.86	0.77
Correlation table for factors:	Factor 1	Factor 2	Factor3
Factor 1	1.00	0.56	0.38
Factor 2	0.56	1.00	0.42
Factor 3	0.38	0.42	1.00

Bold numbers distinguish between factor items

Ordinal alpha was calculated for each item to determine the relative effects on the scale as a whole and the consequence of removing an item on each factor. Ordinal alpha values did not necessitate deletion of any items. Inter item correlations were then investigated between factors and finally the item total correlations for the whole scale, presented in Table 3.

**Table 3:** Item correlations in factors

Items	Corrected item correlation	
	Factor	Scale
Factor 1 “Confidence in provision of care”		
1. I worry about providing care in acute situations	0.71	0.64
2. I trust my ability to provide care in acute situations	0.70	0.64
3. I have sufficient knowledge to provide care in acute situations	0.67	0.64
4. I estimate my general ability to provide care in acute situations	0.73	0.69
5. I estimate my ability to manage the demands that I place upon myself in acute situations	0.77	0.71
6. I estimate my ability to manage demands from my colleagues in acute situations	0.63	0.57
7. I estimate my ability to independently determine necessary actions in acute situations	0.72	0.70
8. I estimate my ability to independently prioritise between actions in acute situations	0.68	0.65
12. I estimate my ability to independently lead bedside care in acute situations	0.69	0.69
16. I estimate my ability to understand the individual patient’s medical needs in acute situations	0.60	0.61
Factor 2 “Communication”		
9. I estimate my ability to take instructions over the telephone in acute situations	0.88	0.69
10. I estimate my ability to carry out instructions that I have received over the phone in acute situations	0.82	0.67
11. I estimate my ability to receive instructions from an attending doctor in acute situations	0.77	0.68
13. I estimate my ability to report a patient’s condition to a nurse in an acute situation	0.70	0.71
Factor 3 “Patient perspective”		
14. I estimate my ability to make patients participate in acute situations	0.79	0.45
15. I estimate my ability to understand the individual patient’s care needs in acute situations	0.77	0.56
17. I estimate my ability to provide information adapted to the needs of the individual in acute situations	0.64	0.55

## Discussion

This study aimed to develop and examine the psychometric properties of a scale that measures novice nurses self-reported perception of their ability to provide care in acute situations. Such a scale could be considered as a useful tool to evaluate the relative effects of initiatives designed to better prepare novice nurses for acute situations and fulfils a need expressed by several authors who have called for new educational initiatives to better prepare nurses for clinical work [9, 12, 15, 39].

The PCAS scale contains 17 items grouped into three factors. The three factors, EFA modeling -” confidence in provision of care” [10 items], 2/ “communication” [4 items] and 3/ “patient perspective” [3 items] are considered relevant to provision of care in acute situations and have been highlighted in previous literature. Porter et al. [40] and Smith et al. [41] have suggested that self-confidence is a key component for clinical performance while improvement in self-confidence for nurses has been suggested as necessary to ensure appropriate care [42]. Effective communication has been indicated by Robertson-Preidler [43] as critical in identifying patient specific needs and risks as well as enabling shared decision making which is important for providing appropriate care. Finally, maintaining a patient perspective is considered a basis of caring science [44].

Item reduction for the PCAS scale was performed using EFA. This method is typically applied in the early stage of scale development as a means of generating hypotheses about underlying processes and consolidating data [29]. There is however, little consensus in the literature regarding decision rules when performing EFA [32]. Our choices are subsequently the result of iterative discussions within the research team with the aim of developing a valid scale that measures novice nurses self-reported perception of ability to care in acute situations.

Validity of the scale was evaluated using cognitive interviewing of novice nurses. Use of the target group to evaluate the scale was important as nobody is more expert in this area than novice nurses themselves [27]. An expert panel of four individuals was also recruited to evaluate content validity of the items. The fact that these experts were independent from the research team is considered a strength of the study [23]. The number of individuals in the expert group is consistent with recommendations [24]. The decision to use a four-point response option to avoid “fence sitting” and force a choice was discussed carefully within the research team and was tested without issue in the cognitive interviews. Given the aim of the scale was to measure self-reported perception of the ability to care and to facilitate the possibility of reflection and discussion over areas in need of improvement, it was considered important that respondents take a stand. One has either the ability to perform or solve a task or not. Indifference was not considered to be a viable option.

Ordinal alpha and Cronbach’s alpha were used to evaluate reliability. While ordinal alpha was considered most appropriate, due to the ordinal nature of the scale, we chose to also report Cronbach’s alpha to allow for comparison with other scales which frequently report this statistic. Both alpha levels were over 0.9. It is commonly accepted that Alpha levels should be at least 0.8 for basic research and 0.9 for clinical instruments [24]. It is important to clarify that ordinal alpha is conceptually equivalent to Cronbach’s alpha though it is performed on polychoric correlations [33].

The PCAS scale is considered to be valid but further investigation is necessary before the scale can be fully implemented. A necessary step will be to trial the scale in clinical practice (i.e. evaluation of interventions and as a guide for reflection), test stability of the factors and establish cut off scores.

As discussed earlier the term ‘acute situations’ is an ambiguous concept [10] and not related to a specific care situation or medical condition [11]. It is also important to recognize that novice nurses can be grouped into different levels of proficiency reflecting their relative experience of providing care in acute situations [7]. We suggest that the PCAS scale could be used to classify novice nurses according to their level of perceived proficiency. This has implications for design of educational initiatives such as simulation-based training which can be customized to the individual nurses’ level of proficiency as well as in relation to those areas perceived as problematic by the novice nurse. Such a simulation would support Benner’s [7] Novice to Expert theory by giving the nurses experience of acute situations and the opportunity to self-reflect over performance and the influences of their decision making.

The authors acknowledge several limitations in the development and testing of the PCAS scale. Despite our best attempts, the number of participants is in the lower range of recommendations for this type of study [23]. One reason for this is that not all regions in Sweden chose to participate in this study. Regional representatives indicated that participation was limited by workload and organizational issues. There were however also regions that did not respond to our enquiry. The number of novice nurses who actually received information about the study is not known as not all hospitals reported the number of nurses invited to participate. It should be recognized that sample size for scale development is restricted by the resources available [23]. This might force a sample size that is adequate but not ideal [28] and it is acknowledged that the model would be less stable because of this.

Another limitation is that the web-based scale required participants to submit their responses by pressing a send button. If the participant did not press this button, no answers was recorded. In the material we have seen that over 90 respondents started the survey but did not submitted it. Reasons for this can only be speculated as data collection was anonymous.

In conclusion, the PCAS scale has proven to have acceptable validity. The factors,” confidence in provision of care”, “communication” and “patient perspective” support previous literature which highlight these areas as important aspects of providing care in acute situations. Additional testing of the PCAS is needed to conclude if it is sensitive enough to evaluate interventions aimed at improving novice nurses competence and suitable as a guide for reflection for novice nurses. The PCAS scale has potential for use in evaluating training interventions for novice nurses and as a basis for discussion and reflection regarding areas in which novice nurses require additional support and training.

## Conclusions

The PCAS scale has proven to have acceptable validity. The factors,” confidence in provision of care”, “communication” and “patient perspective” are likely to be important aspects of providing care in acute situations. Additional testing of the PCAS is needed to conclude if it is sensitive enough to evaluate interventions aimed at improving novice nurses competence and suitable as a guide for reflection for novice nurses.

## Supplementary information

**Additional file 1.** Supplementary table with Item data description.

## Data Availability

The datasets used and/or analysed during the current study available from the corresponding author on reasonable request.

## Abbreviations

EFA: Exploratory factor analysis
KMO: Kaiser-Meyer-Olkin
PCAS: Perception to Care in Acute Situations scale
