# Supplementary material for: Development and initial validation of an instrument to measure novice nurses’ perceived ability to provide care in acute situations – PCAS
Source: BMC Nurs. 2020 Feb 17;19:13. doi: 10.1186/s12912-020-0406-3 (PMC7027289; doi:10.1186/s12912-020-0406-3)
Supplement: Supplementary file 1 — Additional file 1. Supplementary table with Item data description. [file 12912_2020_406_MOESM1_ESM.docx]

Supplementary table with Item data description
